# Coordination of emergency resources after Lorca's earthquakes

**DOI:** 10.1186/cc11090

**Published:** 2012-03-20

**Authors:** L Escobar, A Ferrández, J Jimenez, A Peláez, A Corbatón, R Alvaro

**Affiliations:** 1 Hospital Rafael Méndez, Lorca, Spain; 2 Gerencia 061, Murcia, Spain; 3 Hospital Clínico San Carlos, Madrid, Spain; 4 Área II Servicio Cantabro de Salud, Santander, Spain

## Introduction

This work's purpose is to describe the coordination of different medical resources after Lorca's 2011 earthquakes. They caused 11 deaths, including two pregnant women and their babies, many injured, moderate or severe damage to 80% of the buildings, and more than 30,000 people without shelter.

## Methods

A descriptive study of the files of Murcia's Emergency Coordination Center (ECC) on the activation of resources after the earthquakes.

## Results

**Figure 1:**
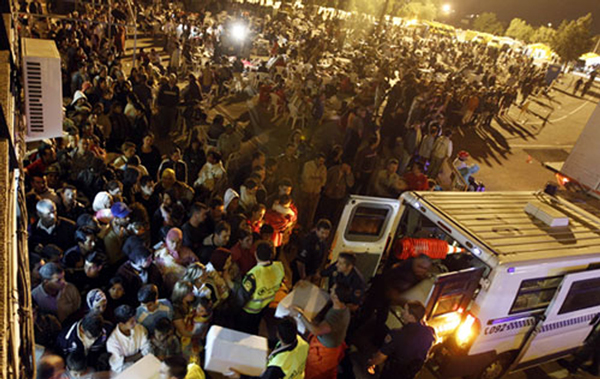
**Thirty thousand people need shelter after the earthquakes**.

Time 17:06 hours: first call. Local resources and city emergency plan are activated. Four medical teams (UME) are pre-activated. 18:49 hours: incoming calls alert of buildings crumbling, dead among the rubble, and hundreds of injured. 18:55 hours: seven UME from five cities are sent to Lorca. 19:00 hours: telephone communications collapse. The ECC uses its internal network. An Advanced Command Point (ACP) is established with a field hospital. 19:10 hours: Rafael Mendez Hospital (225 patients) has to be evacuated. Medical personnel of the hospital, private ambulances and UMEs begin the evacuation. The emergency service of the hospital continues to be operative in the building until evacuation is completed and in a field hospital later. 19:20 hours: the Military Emergencies Unit is required for activation. 19:30 hours: the military and emergency services field hospitals are sent to Lorca. 19:50 hours: Virgen del Alcázar Hospital has to be evacuated (145 patients). 20:25 hours: at the ACP field hospital of the Red Cross, Civil Protection and Emergency Services are being set. 20:30 hours: 11 hospitals in six provinces are contacted to relocate evacuated patients. 20:40 hours: all buildings in Lorca are have been evacuated. Thirty thousand people need shelter. Ten camps with tents are set throughout Lorca by the Red Cross, Emergency Services and Civil Protection to give shelter to 16,000 people. See Figure 1.

## Conclusion

Coordination of the different medical and emergency services by the ECC made possible correct use of resources and fast attention to the population that minimized the effects of the catastrophe.

